# Investigation of the anti-inflammatory effects of native potential probiotics as supplementary therapeutic agents in an in-vitro model of inflammation

**DOI:** 10.1186/s12906-023-04153-y

**Published:** 2023-09-21

**Authors:** Shadi Aghamohammad, Amin Sepehr, Seyedeh Tina Miri, Saeideh Najafi, Mohammad R. Pourshafie, Mahdi Rohani

**Affiliations:** 1 Department of Bacteriology, Pasteur Institute of Iran, Tehran, Iran; 2 Department of Biology, Science and Research Branch, Islamic Azad University, Tehran, Iran

**Keywords:** Signaling pathway, Inflammatory bowel disease, Probiotic, *Lactobacillus*, *Bifidobacterium*

## Abstract

**Background:**

IBD is considered an inflammatory disease with abnormal and exaggerated immune responses. To control the symptoms, different theraputic agents could be used, however, utilizing the agents with the least side effects could be important. Probiotics as beneficial microorganisms are one of the complementory theraputic agents that could be used to modulate inflammatory signaling pathways. In the current study, we aimed to identify the precise molecular effects of potential probiotics on signaling pathways involved in the development of inflammation.

**Methods:**

A quantitative real-time polymerase chain reaction (qPCR) assay was used to analyze the expression of JAK /STAT (*JAK1, JAK2, JAK3, TYK2, STAT1, STAT2, STAT3, STAT4, STAT5* and *STAT6*) and inflammatory genes (*NEMO*, *TIRAP*, *IRAK*, and *RIP*) after the HT -29 cell line treatment with the sonicated pathogens and potential probiotics. A cytokine assay was also used to evaluate IL -6 and IL -1β production after potential probiotic treatment.

**Results:**

The potential probiotic cocktail downregulated the *JAK* genes and *TIRAP*, *IRAK4*, *NEMO*, and *RIP* genes in the NF-kB pathway compared with cells that were treated with sonicated gram negative pathogens. The expression of *STAT* genes was different after potential probiotic treatment. The production of IL -6 and IL -1β decreased after potential probiotic treatment.

**Conclusions:**

Considering the importance of controlling the symptoms of IBD to improve the life quality of the patients, using probiotic could be crucial. In the current study the studied native potential probiotic cocktails showed anti-inflammatory effects via modulation of JAK /STAT and NF-kB signaling pathways. This observation suggests that our native potential probiotics consumption could be useful in reducing intestinal inflammation.

## Background

The inflammatory response is a mechanism that plays a role in the neutralization of pathogens. Any alteration in immunological mechanisms leading to excessive activity of the inflammatory process could be considered a causative factor for inflammatory diseases, including inflammatory bowel disease (IBD) [1]. IBD is an inflammatory disease with the course of remission and relapse. The disease is divided into two types, ulcerative colitis and Crohn’s disease, with common but also different symptoms [2]. Given that the treatment symptoms of these two types can occasionally exhibit dissimilarities, it is imperative to have precise diagnosis using biomarkers. Undoubtedly, the diagnosis of IBD is considered as an important matter in order to better control and management of the disease. IBD can be observed in various regions across the globe, encompassing Europe, Africa, and South America, and predominantly affecting youthful individuals [3].Several factors, including stress, smoking, genetic susceptibility, diet, and microbiome, are involved in the development of IBD [4]. In the mild and moderate forms of the disease, the use of chemical agents is usually recommended, but in severe IBD, surgical strategies are usually required [5]. Using the treatments with the least side effects could be critical for patients involved with IBD.

NF-kB (Nuclear Factor kappa-light-chain-enhancer of activated B cells) is one of the important signaling pathways involved in IBD. Under normal conditions, the activity of the NF-kB pathway is low, however, the expression of several genes involved in this pathway might be increased in the inflammatory state [6]. Several genes, are involved in the NF-kB signaling pathway, that their expression may be affected in IBD [7]. Another signaling pathway that may play a role in the development of IBD is the Janus kinase/signal transduction and activator of transcription (JAK /STAT) pathway. Each component of this signaling pathway can be associated with different types of cytokines. For example, IL -10 and other family members (IL -19, IL -20, IL -22, and IL -26) for instance, share signaling through JAK1, JAK2, TYK2, and STAT3. Interfering with the components of this signaling pathway could be a potential treatment to control IBD [8].

Probiotics are beneficial microorganisms that have recently been shown to be potentially effective in alleviating the symptoms of IBD and reducing inflammation. Probiotics could have beneficial effects via influencing several mechanisms [9]. Dysbiosis and imbalance of normal intestinal flora are common in patients with IBD [10]. Balancing the intestinal normal flora and modulating inflammation by influencing the immune system and inflammatory signaling pathways are some of the beneficial effects of probiotics in alleviating patients’ symptoms and prolonging stable conditions [10, 11]. *Lactobacillus* spp. *and Bifidobacterium* spp. are two types of probiotics with beneficial effects on modulating inflammation. These species are found in commercially available probiotic preparations used to control and treat IBD. The immunomodulatory effects of each species could be different. Therefore, evaluating the exact mechanism of the probiotic species could be useful to choose the appropriate strategies [12].

The phenotypic effects of our native probiotics on the control of inflammatory status have already been observed [13]. Identifying the precise molecular effects of probiotics on signaling pathways involved in the development of inflammation could reveal the putative beneficial effects of probiotics. Therefore, in the present study, we aimed to investigate the efficacy of our native potential probiotic strains in modulating JAK /STAT and regulating inflammatory signaling pathways to understand how these potential probiotics, reduce the inflammation.

## Methods

### Bacterial strains and treatment of HT-29 cells with potential probiotics and sonicated pathogens

In this study, the in-vitro assay was performed to evaluate the effects of potential probiotics on the NF-kB and JAK/STAT signaling pathways after inflammation induction. To trigger the inflammation, the sonicated pathogens were used. The potential probiotic and phenotypic characteristics of these probiotic strains, including four *Lactobacillus* spp. and three *Bifidobacterium* spp. were previously studied [13, 14]. The culture process of potential probiotic strains and pathogenic bacteria, including sonicated enterotoxin-producing *Escherichia coli* (ETEC) and *Salmonella typhimurium*, along with the cell culture process had been described, previously [15]. All methods were performed according to the relevant guidelines and regulations, and ethical approval for the previous study was obtained from the committee of the Pasteur Institute of Iran (IR.PII. REC.1398.060). Signed informed consent was obtained from all participants.

### Sonicated pathogen and potential probiotics treatments

HT -29 cells were exposed to various bacteria, either alone or as mixtures, including sonicated enterotoxigenic *E. coli* (SP-ETEC), sonicated *Salmonella typhi* (SP-ST), *Lactobacillus* spp. alone, *Bifidobacterium* spp. alone, *Lactobacillus*/*Bifidobacterium* mixture (Lac/Bif). To evaluate the effects of potential probiotics on inflammation, different treatments of HT -29 cells were performed. The treatments of HT -29 cells were performed as follows: First, SP-ETEC and SP-ST were added to the HT -29 cell line to induce inflammation. Second, *Lactobacillus* spp., *Bifidobacterium* spp., and Lac/Bif were added after 6 h to determine the presumed effects. After 1 h, each well was washed twice with PBS to remove the non-adherent bacteria. These treatments were performed in duplicate and the cell culture was maintained at 37 °C and 5% CO2 for up to 48 h. Determination of MOI was performed as previously indicated [16].

### Cytokine assays

To evaluate the phenotypic results of potential probiotic treatment on the reduction of inflammation, cytokine production was assessed by ELISA assay. This step was performed after treatment of HT -29 cells with potential probiotics. The supernatant of the cell culture was centrifuged at 6000 rpm, and the supernatant was collected to evaluate the production of pro-inflammatory cytokines, including IL -6 and IL -1β.

### RT- PCR of inflammatory signaling pathway genes

RNA extraction kit (Roche, Germany) was used to extract total RNA according to the manufacturer’s instructions. The quantity and quality of the purified RNA were determined by a NanoDrop1000 UV-Vis Spectrophotometer (measuring the absorbance at 260/280 nm). The cDNA template was synthesized with the cDNA synthesis kit (Yekta Tajhiz, Iran) according to the manufacturer’s instructions. The online Primer-Bank website (http://pga.mgh.harvard.edu/primerbank) was used to choose the qPCR primers (Table 1). All the reactions were performed in duplicate. The formula RQ = 2^−ΔΔCt^ was used to get relative gene expression in the comparative CT method [17]. The appropriate internal control gene, glyceraldehyde 3-phosphate dehydrogenase (*gapdh*), was selected as a housekeeping gene to normalize the data. ABI step one plus detection system (Applied Biosystems, USA co) and SYBR Green master mix (Amplicon Bio, Denmark) was used to evaluating the mRNA quantification of studied genes.

**Table 1:** Primer sequences used in this study

Gene	Primer Sequence [5’ > 3’]	Primer Bank ID	Product Size [bp]
STAT1 F STAT1 R	CGGCTGAATTTCGGCACCT CAGTAACGATGAGAGGACCCT	189458859c3	81
STAT2 F STAT2 R	CTGCTAGGCCGATTAACTACCC TCTGATGCAGGCTTTTTGCTG	291219923c3	87
STAT3 F STAT3 R	ACCAGCAGTATAGCCGCTTC GCCACAATCCGGGCAATCT	47080104c2	124
STAT4 F STAT4 R	GCTTAACAGCCTCGATTTCAAGA GAGCATGGTGTTCATTAACAGGT	345110659c2	91
STAT5 F STAT5 R	CGACGGGACCTTCTTGTTG GTTCCGGGGAGTCAAACTTCC	221316717c3	80
STAT6 F STAT6 R	CGAGTAGGGGAGATCCACCTT GCAGGAGTTTCTATCAAGCTGTG	296010867c2	92
JAK1 F JAK1 R	CTTTGCCCTGTATGACGAGAAC ACCTCATCCGGTAGTGGAGC	102469033c1	101
JAK2 F JAK2 R	ATCCACCCAACCATGTCTTCC ATTCCATGCCGATAGGCTCTG	223671934c2	121
JAK3 F JAK3 R	CTGCACGTAGATGGGGTGG CACGATCAGGTTGGACTTTTCT	189095272c2	78
TYK2 F TYK2 R	GAGATGCAAGCCTGATGCTAT GGTTCCCGAGGATTCATGCC	187608614c1	76
RIP2 F RIP2 R	GCCCTTGGTGTAAATTACCTGC GGACATCATGCGCCACTTT	93141034c2	138
NEMO F NEMO R	AAGAGCCAACTGTGTGAGATG TTCGCCCAGTACGTCCTGA	142381344c1	69
TIRAP F TIRAP R	GACCCCTGGTGCAAGTACC CGACGTAGTACATGAATCGGAG	89111123c2	133
IRAK4 F IRAK4 R	CTTGGATGGTACTCCACCACT AAAATTGATGCCATTAGCTGCAC	223671887c3	76

### Statistical analysis

Graphs and statistical analysis of the data were performed using SPSS (ver.25) and GraphPad Prism software to compare variables of different groups. Statistical differences between multiple groups, including control (C), sonicated pathogen (SP), first pathogen, and then *Lactobacillus* spp. was given (PL), the first pathogen, and then *Bifidobacterium* spp. (PB), and first pathogen and then Lac/Bif was given (PLB), were determined using ordinary one-way ANOVA. *P-values <* 0.05 were considered statistically significant. The results were presented as Standard Deviation (SD).

## Results

The effectiveness of potential probiotics in up or downregulation of the studied genes was examined by comparison between treated HT-29 cells with potential probiotics versus control cells (not exposed HT-29 cells as a negative control, i.e. C24 and C48) and HT-29 cells exposed to the sonicated pathogen as a positive control (i.e. SP24 and SP48).

### The effects of potential probiotic strains on the expression of *STAT* genes

**Fig. 1:**
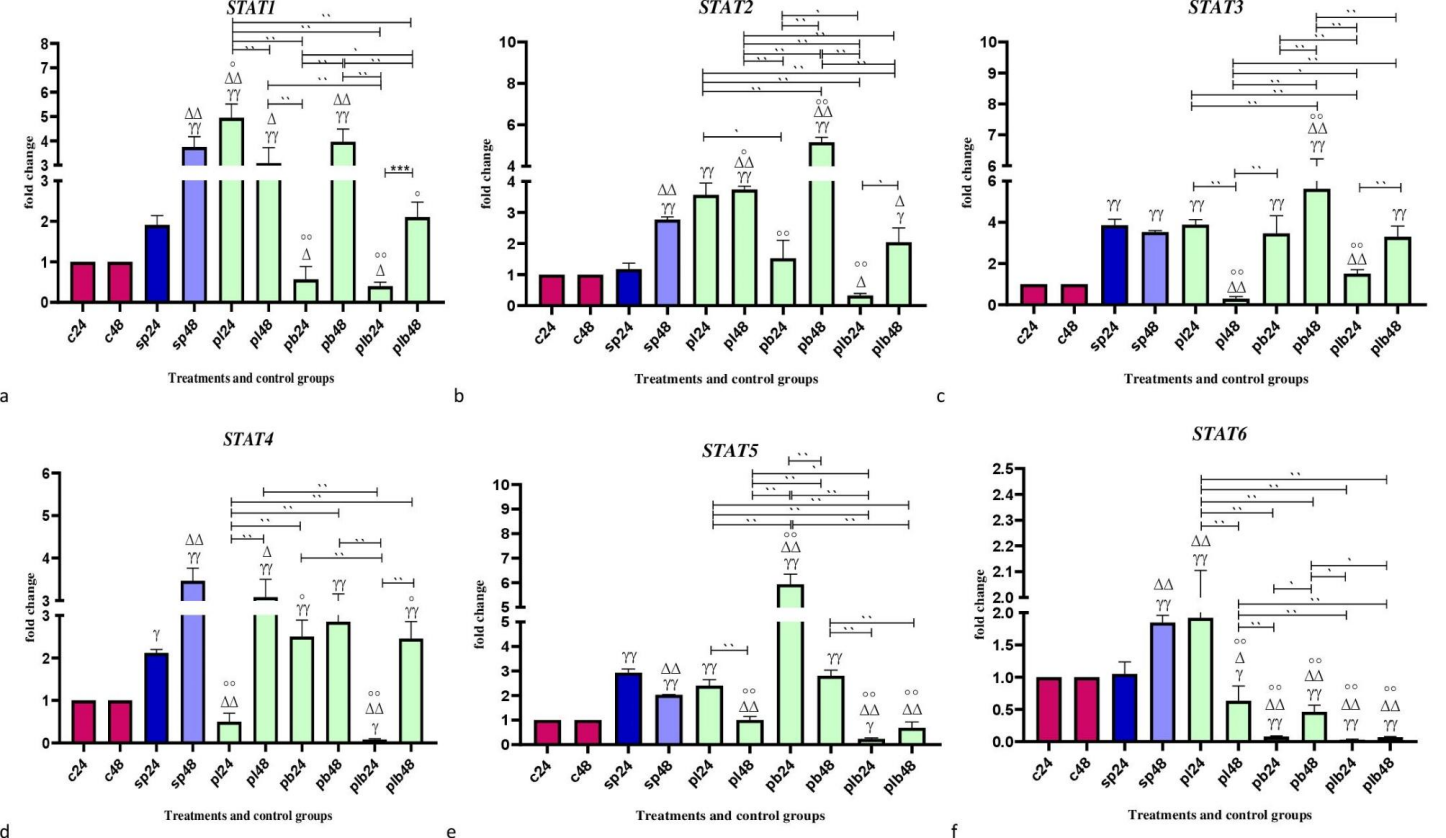
Relative gene expression (mean fold change) of (**a**) *STAT1*, (**b**) *STAT2*, (**c**) *STAT3*, (**d**) *STAT4*, (**e**) *STAT5*, and (**f**) *STAT6* in the different groups of treatments. Data were normalized with *gapdh*. Data were represented as mean SD. The number 24 and 48 refers to different time orders of HT-29 cell line treatments. **C**, control; **SP**, Sonicated Pathogen; **PL**, first sonicated pathogen and then *Lactobacillus* spp.; **PB**, first pathogen and then *Bifidobacterium* spp.; **PLB**, first pathogen and then Lac/Bif. Data were considered as statistically significant when p < 0.05 (*p < 0.05, **p < 0.001). The **γ** symbol indicates the relatedness between C24 and C48 with other treatments, the **Δ** symbol shows the relatedness between P24 and other treatments, and the empty circle shows the relatedness between SP48 with other treatments. The relatedness between other treatments is shown with brackets

Data on *STAT* gene expression are shown in Fig. 1. Comparative analysis of the STAT genes showed different changes in gene expression levels. Some of the potential probiotic treatments significantly increased the gene expression, while the others decreased the expression. The comparative analysis of STAT gene expression between the sonicated pathogens and the negative controls showed that SP-ETEC and SP-ST could significantly increase the gene expression, especially after 48 h.

In *STAT1*, *Lactobacillus* spp. and *Bifidobacterium* spp. had opposite effects on gene expression. *Lactobacillus* spp. had the strongest effect on upregulating expression after 24 h of treatment (PL24) compared to negative controls (p < 0.001) and positive control (p < 0.05). On the other hand, *Bifidobacterium* spp. and Lac/Bif had the most significant effects on reducing the expression level in the first 24 h of treatment (PB24 and PLB24) (p < 0.05).

All formats of potential probiotics were able to significantly increase *STAT2* gene expression at 48 h. *Bifidobacterium* spp. at 48 h (PB48) could increase the expression level more than other potential probiotic treatments (p < 0.001). On the other hand, Lac/Bif at the first 24 h (PLB24) had the strongest down-regulatory effect (p < 0.05).

Comparative analysis of *STAT3* gene revealed the opposite effects of *Lactobacillus* spp. and *Bifidobacterium* spp. *Lactobacillus* spp. decreased the expression level after 48 h (PL48), while *Bifidobacterium* spp. upregulated the gene expression (PB48).

In *STAT4*, it can be said that the general trend of gene expression was downward. Most treatments were able to decrease gene expression, especially compared to SP48, except *Lactobacillus* spp. (PL48), which was able to significantly upregulate gene expression compared to SP24. *Lactobacillus* spp. and Lac/Bif at 24 h (PL24 and PLB24) had the most significant effects (p < 0.001).

Comparative analysis of *STAT5* gene showed that *Lactobacillus* spp. (PL48) and Lac/Bif (PLB24 and PLB48) could decrease the expression level (p < 0.001), while *Bifidobacterium* spp. (PB24 and PB48) could significantly upregulate the gene expression (P < 0.001).

For *STAT6*, it can be said that the overall trend of gene expression was downward. All potential probiotic treatments decreased the expression level, except for *Lactobacillus* spp. (PL24). The expression level was close to zero for Lac/Bif treatments at both time points (PLB24 and PLB48).

### The effects of potential probiotic strains on the expression of *JAK* genes

**Fig. 2:**
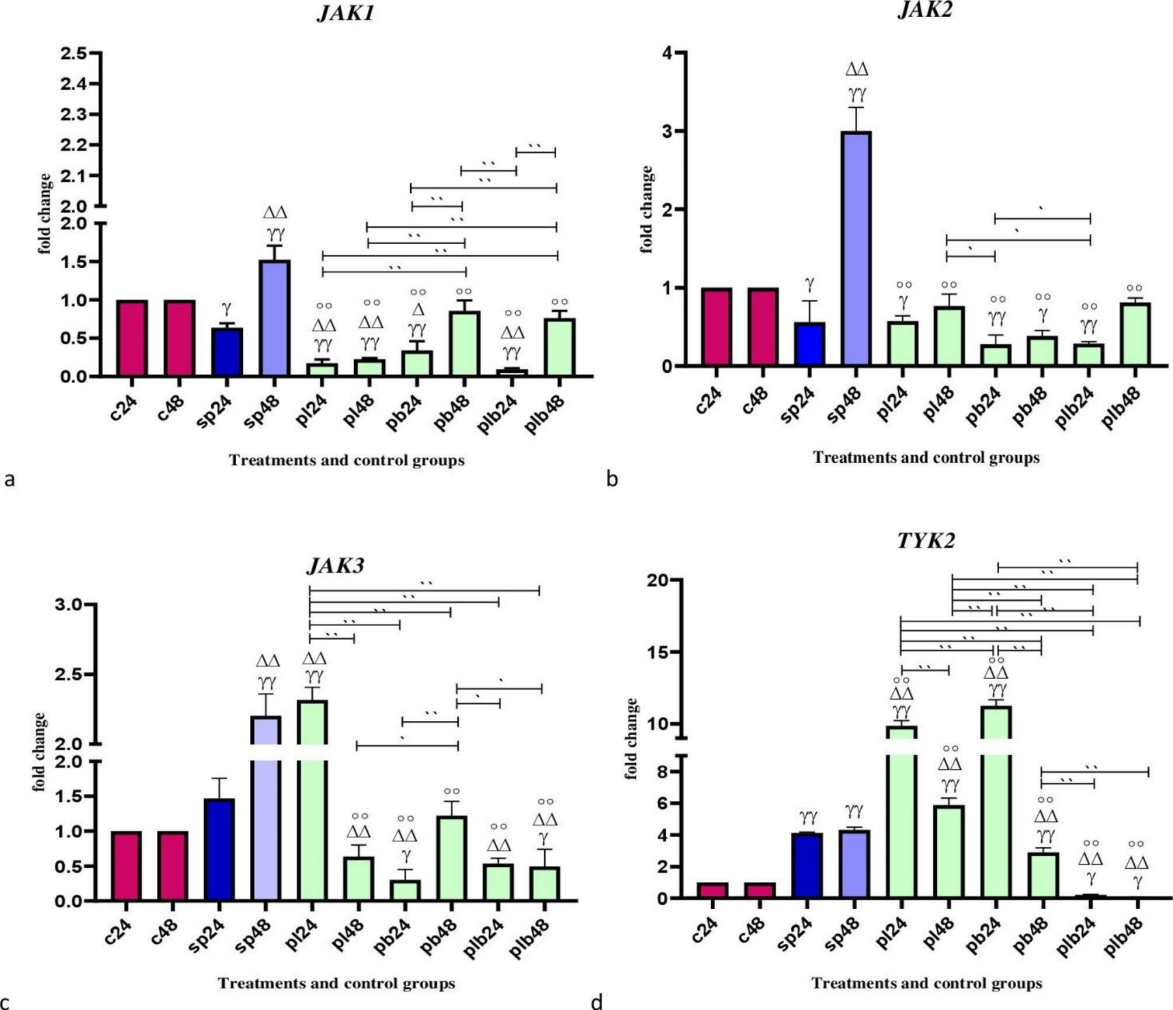
Relative gene expression [mean fold change] of (**a**) *JAK1*, (**b**) *JAK2*, (**c**) *JAK3*, and (**d**) *TYK2* in the different groups of treatments. Data were normalized with *gapdh*. Data were represented as mean SD. The number 24 and 48 refers to different time orders of HT-29 cell line treatments. **C**, control; **SP**, Sonicated Pathogen; **PL**, first sonicated pathogen and then *Lactobacillus* spp.; **PB**, first pathogen and then *Bifidobacterium* spp.; **PLB**, first pathogen and then Lac/Bif. Data were considered as statistically significant when p < 0.05 (*p < 0.05, **p < 0.001). The γ symbol indicates the relatedness between C24 and C48 with other treatments, the Δ symbol shows the relatedness between P24 and other treatments, and the empty circle shows the relatedness between SP48 with other treatments. The relatedness between other treatments is shown with brackets

Data on *JAK* expressions are shown in Fig. 2. Comparative analysis of *JAK* gene expression between sonicated pathogens and negative control showed that SP-ETEC and SP-ST could significantly increase gene expression.

Comparative analysis of *JAK1* and *JAK2* showed a downward trend in expression. All potential probiotic treatments could significantly decrease the expression level, especially compared to SP48 (p < 0.001).

For *JAK3*, all potential probiotic treatments were able to significantly downregulate the expression level compared to SP48 (p < 0.001), with the exception of PL24.

Comparative analysis of *TYK2* showed different changes in gene expression levels. *Lactobacillus* spp. in both time sequences (PL24 and PL48) and *Bifidobacterium* spp. in the first 24 h of treatment increased the expression level (p < 0.001), while Lac/Bif (PLB24 and PLB48) together with *Bifidobacterium* spp. after 48 h (PB48) were able to downregulate gene expression (p < 0.001).

### The effects of potential probiotic strains on the expression of the inflammatory genes

**Fig. 3:**
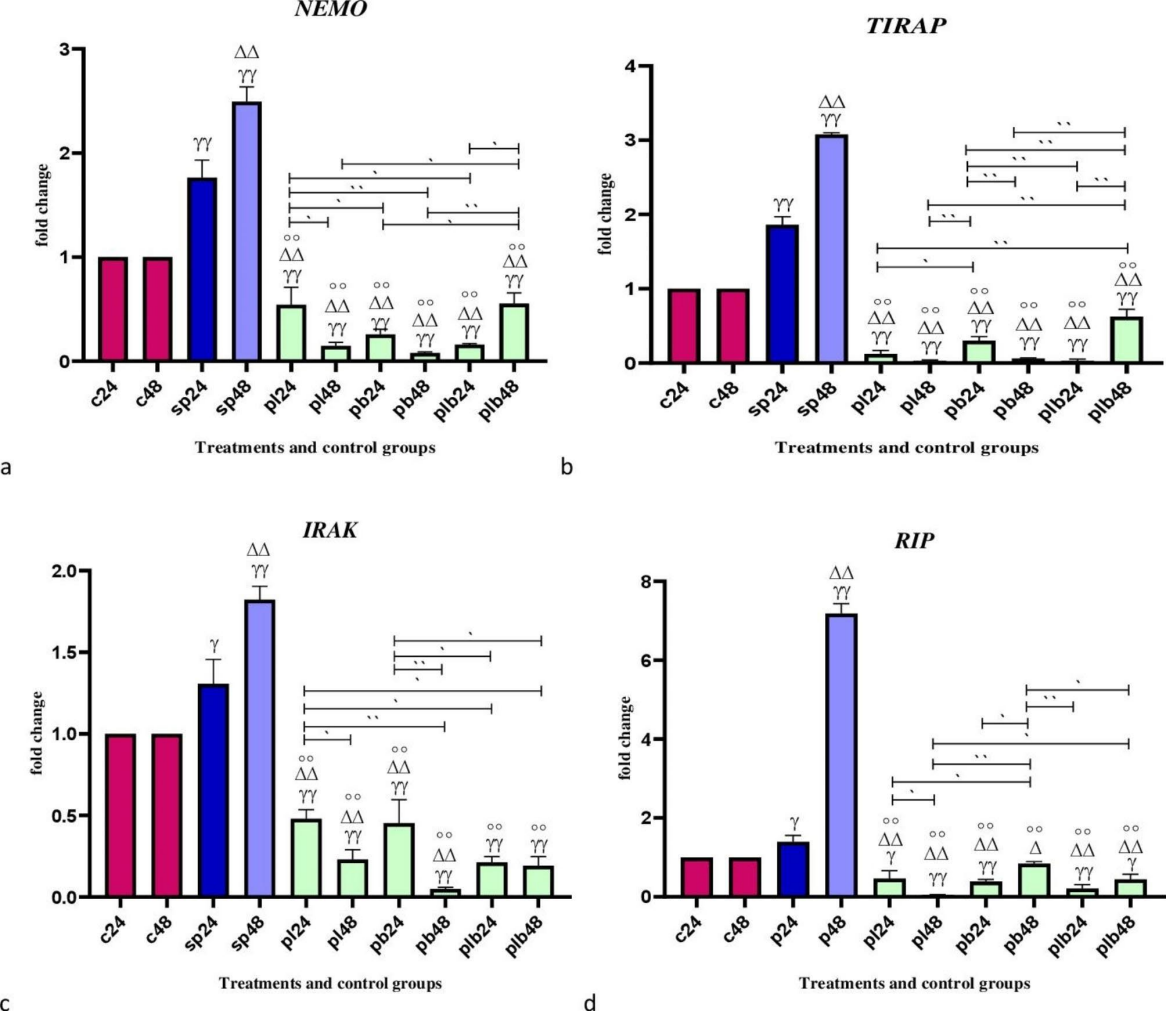
Relative gene expression [mean fold change] of (**a**) *NEMO*, (**b**) *TIRAP*, (**c**) *IRAK*, and (**d**) *RIP* in the different groups of treatments. Data were normalized with *gapdh*. Data were represented as mean SD. The number 24 and 48 refers to different time orders of HT-29 cell line treatments. **C**, control; S**P**, Sonicated Pathogen; **PL**, first sonicated pathogen and then *Lactobacillus* spp.; **PB**, first pathogen and then *Bifidobacterium* spp.; **PLB**, first pathogen and then Lac/Bif. Data were considered as statistically significant when p < 0.05 (*p < 0.05, **p < 0.001). The γ symbol indicates the relatedness between C24 and C48 with other treatments, the Δ symbol shows the relatedness between P24 and other treatments, and the empty circle shows the relatedness between SP48 with other treatments. The relatedness between other treatments is shown with brackets

Inflammatory gene expression data are shown in Fig. 3. Comparative analysis of inflammatory gene expression, including *NEMO*, *TIRAP*, *IRAK*, and *RIP* between sonicated pathogens and negative controls showed that SP-ETEC and SP-ST could significantly increase gene expression, especially after 48 h. A downward trend in gene expression was observed for all studied genes and all potential probiotic treatments down-regulated inflammatory genes (p < 0.001). There was no significant difference between *Bifidobacterium* spp, *Lactobacillus* spp and Lac/Bif in decreasing the expression level of inflammatory genes.

### The result of pro-inflammatory cytokines production

**Fig. 4:**
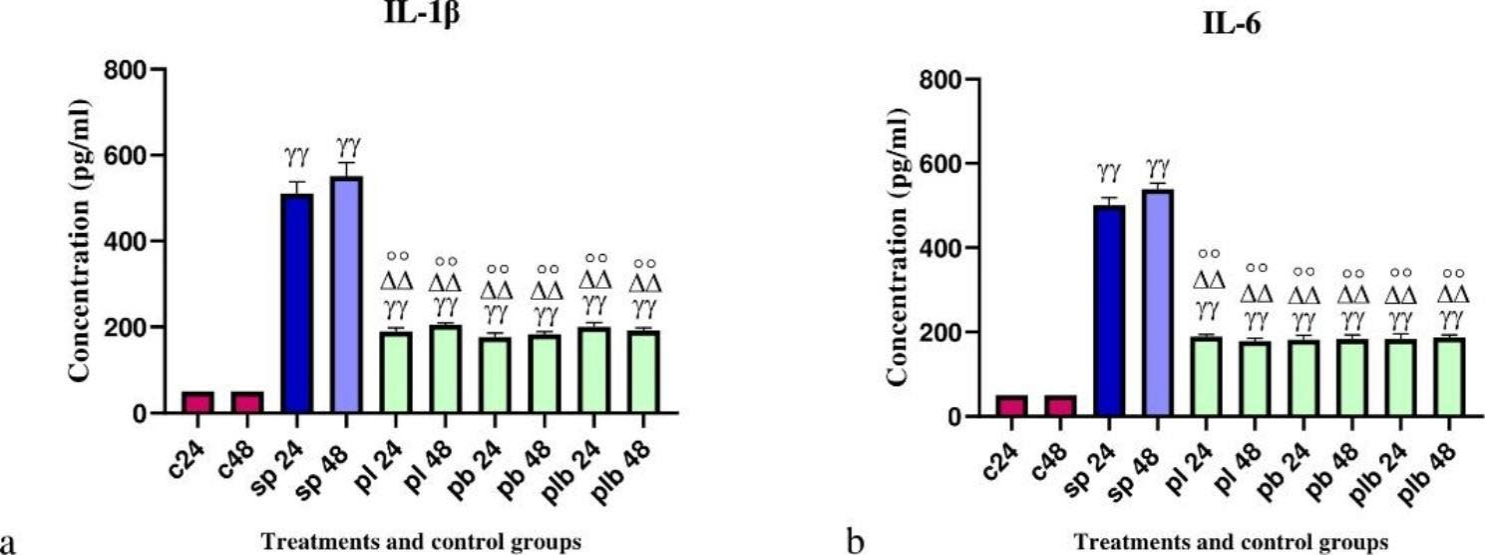
Different levels of concentrations of (**a**) IL-1β and (**b**) IL-6. Data were represented as mean SD. The number 24 and 48 refers to different time orders of HT-29 cell line treatments. **C**, control; S**P**, Sonicated Pathogen; **PL**, first sonicated pathogen and then *Lactobacillus* spp.; **PB**, first pathogen and then *Bifidobacterium* spp.; **PLB**, first pathogen and then Lac/Bif. Data were considered as statistically significant when p < 0.05 (*p < 0.05, **p < 0.001). The γ symbol indicates the relatedness between C24 and C48 with other treatments, the Δ symbol shows the relatedness between P24 and other treatments, and the empty circle shows the relatedness between SP48 with other treatments

The results of pro-inflammatory cytokines production are shown in Fig. 4. Cytokine production was significantly higher after SP treatments. However, potential probiotic treatments (6 h after SP treatment) significantly decreased cytokine production. No significant difference was observed between *Lactobacillus* spp., *Bifidobacterium* spp. and Lac/Bif at any time point after the treatments.

## Discussion

IBD is a chronic relapsing inflammatory disease with a low mortality rate, although the choice of appropriate strategies leading to a reduction of symptoms could impressively improve the life quality of IBD patients [18]. The use of nonpharmacologic options, including probiotics, is assumed as a complementary treatment to manage symptoms in patients with IBD [19]. Assessing the precise molecular effects of probiotics on signaling pathways may be useful to clarify the role of probiotics in controlling IBD. To achieve this goal, we investigated NF-kB and JAK /STAT signaling pathways after potential probiotic treatment of the HT -29 cell line and decided to use potential probiotics as post-treatment (6 h after SP -treatment) to evaluate the role of potential probiotics as complementary treatment options.

In the present study, a comparative analysis of *STAT* genes showed different changes in geneexpression levels. Both down- and up-regulation of gene expression was observed in *STAT1*, *STAT2*, *STAT3*, and *STAT5* after potential probiotic treatment. *Bifidobacterium* spp. was generally able to increase the expression level of the mentioned genes after 48 h of treatment, while Lac/Bif had the opposite effect and downregulated the expression level. Both a decrease and an increase in the expression level were observed with *Lactobacillus* spp. treatment. For *STAT4* and *STAT6*, the overall trend of gene expression was downward. These observations are confirmed by other studies. Guo et al. have postulated that the utilization of probiotic strains, specifically *Bifidobacterium pseudolongum*, may exhibit a noteworthy impact in the mitigation of colitis in an in-vivo model. This effect is achieved by a decrease in the expression of colonic MyD88 and NF-κB, as well as an increase in the expression of STAT3, Nrf2, and PPARγ [20]. Furthermore, Wu et al., reported that the administration of a high dose of *Bifidobacterium animalis* A6 resulted in a noteworthy reduction in STAT6 expression levels in comparison to DSS-treated BALB/c mice [21]. These effects may potentially be exerted through a multitude of reasons. As mentioned above, the JAK /STAT pathway plays several roles in the context of cytokine functions [22]. However, the exact changes in the functions of STATs during IBD are complicated. STAT1 and STAT3, for example, on the one hand, are associated with some of the anti-inflammatory cytokines, including IL -21 [23]. IL -21 was able to induce significantly higher levels of the cytokine IL -10 and act as an anti-inflammatory agent [24]. Thus, it seems that the upregulation of *STAT1* and *STAT3* might be associated with anti-inflammatory functions, which are an appropriate consequence of IBD and lead to improvement. On the other hand, some researchers reported that the activation of *STAT1* and *STAT3* could lead to the promotion of inflammation and therefore be involved in the pathogenesis of UC, and CD [22]. The same status could be observed for STAT5. While upregulation of STAT5 was observed in the colon of patients with IBD, deletion of STAT5 was associated with dysregulation of the immune system, which was observed in CD [25]. Besides, studies show that IL-22, as both a pro and anti-inflammatory cytokine, could promote the activation of STAT1, STAT3, and STAT5 [26, 27]. Consequently, it appears that some of the components of STAT, including STAT1, STAT3, and STAT5, are associated with different types of cytokines and may play dual roles that ultimately lead to control of inflammation via control of cytokine functions. In contrast, the functions of the other components appear to be more homogeneous. Experimental models have shown that deletion of STAT4 may lead to protection against the development of colitis [28]. In addition, STAT6 is associated with IL -4 and IL -13. As mentioned above, IBD is classified as an autoimmune disease, and regulation of the proinflammatory activity of cytokines by anti-inflammatory and immunosuppressive cytokines such as IL -4 and IL-10 may be helpful in controlling the severity of such diseases [29, 30].

Concerning *JAK* expression, our results were more homogeneous. Our potential probiotics downregulated the expression of JAK genes. The development of molecules that act as JAK inhibitors may be a promising strategy for the treatment of IBD. The use of JAK inhibitors could lead to targeting JAKs and reducing IL -6 production [31]. Recently, many studies have been conducted to find new JAK inhibitors [32]. In the present study, the potential probiotic strains were able to act as JAK inhibitors by reducing the expression level. Overall, all potential probiotic treatments had similar effects on reducing expression levels. However, when all JAK genes are considered, the effect of Lac/Bif could be more substantial.

Here, in addition to the JAK /STAT pathway, the effect of potential probiotics on the NF-kB signaling pathway was investigated. The increased expression of inflammatory genes by sonicated pathogens demonstrated the role of pathogens in the development of inflammation. However, our potential probiotic treatments had a remarkable reducing effect. There was no significant difference between *Bifidobacterium* spp., *Lactobacillus* spp., and Lac/Bif in reducing the expression of inflammatory genes. It should be noted that the potential probiotic treatments reduced the expression of *TIRAP* and *RIP* to zero and all three versions of the potential probiotic treatments were able to have this remarkable effect. Other studies also approve the findings of the current study. According to Gao et al., using *Lactobacillus rhamnosus* GG could have mitigated affects on NF-Kb and MAPK signalling pathways in compared to the cells that were treated with LPS [33]. These genes play an important role in inflammatory signaling pathways, so reducing their expression could lead to a reduction in the production of pro-inflammatory cytokines [34].

## Conclusion

In conclusion, in this study, native potential probiotic bacteria, including *Lactobacillus* and *Bifidobacterium* were used as separate and combined cocktails to evaluate the amelioration of inflammatory effects. Despite the hypothesis that the use of a variety of bacteria in the probiotic diet may help to enhance the beneficial effects, this study demonstrated that our native potential probiotic cocktails in all types, could play anti-inflammatory role in ameliorating the inflammation by affecting the JAK/STAT and NF-kB signaling pathways. Since IBD is a chronic and recurrent inflammatory disease, the use of our native potential probiotics with the least side effects could be suitable to improve the health quality of patients and could be so important to find the easiest way to improve the life quality of IBD patients.

## Data Availability

The datasets generated during and/or analyzed during the current study are available from the corresponding author on reasonable request.

## Declarations

### Abbreviations

IBD, inflammatory bowel disease; JAK, janus kinase; STAT, signal transducer and activator of transcription; NF-кB, Nuclear Factor kappa-light-chain-enhancer of activated B cells; TIRAP, TIR domain-containing adaptor protein; IRAK, IL -1 receptor-associated kinase; RIP, receptor-interacting serine/threonine-protein kinase 2; NEMO, NF-kappa-B essential modulator; qPCR, quantitative real-time polymerase chain reaction; ETEC, enterotoxin-producing Escherichia coli; SP-ETEC, sonicated enterotoxigenic *E. coli*; SP-ST, sonicated *Salmonella typhi*; Lac/Bif, *Lactobacillus*/*Bifidobacterium* mixture; PL, first sonicated pathogen and then *Lactobacillus* spp.; PB, first pathogen and then *Bifidobacterium* spp.; PLB, first pathogen and then Lac/Bif; SD, Standard Deviation.
